# Gasdermin D Deficiency Limits the Transition of Atherosclerotic Plaques to an Inflammatory Phenotype in *ApoE* Knock-Out Mice

**DOI:** 10.3390/biomedicines10051171

**Published:** 2022-05-19

**Authors:** Pauline Puylaert, Melissa Van Praet, Frederik Vaes, Cédric H. G. Neutel, Lynn Roth, Pieter-Jan Guns, Guido R. Y. De Meyer, Wim Martinet

**Affiliations:** Laboratory of Physiopharmacology, University of Antwerp, 2610 Antwerp, Belgium; pauline.puylaert@uantwerpen.be (P.P.); melissa.vanpraet@uantwerpen.be (M.V.P.); frederik.vaes@student.uantwerpen.be (F.V.); cedric.neutel@uantwerpen.be (C.H.G.N.); lynn.roth@uantwerpen.be (L.R.); pieter-jan.guns@uantwerpen.be (P.-J.G.); guido.demeyer@uantwerpen.be (G.R.Y.D.M.)

**Keywords:** gasdermin D, pyroptosis, inflammation, atherosclerosis

## Abstract

Gasdermin D (GSDMD) is the key executor of pyroptotic cell death. Recent studies suggest that GSDMD-mediated pyroptosis is involved in atherosclerotic plaque destabilization. We report that cleaved GSDMD is expressed in macrophage- and smooth muscle cell-rich areas of human plaques. To determine the effects of GSDMD deficiency on atherogenesis, *ApoE^−/−^ Gsdmd*^−/−^ (*n* = 16) and *ApoE^−/−^* *Gsdmd^+/+^* (*n* = 18) mice were fed a western-type diet for 16 weeks. Plaque initiation and formation of stable proximal aortic plaques were not altered. However, plaques in the brachiocephalic artery (representing more advanced lesions compared to aortic plaques) of *ApoE^−/−^ Gsdmd^−/−^* mice were significantly smaller (115 ± 18 vs. 186 ± 16 × 10^3^ µm^2^, *p* = 0.006) and showed features of increased stability, such as decreased necrotic core area (19 ± 4 vs. 37 ± 7 × 10^3^ µm^2^, *p* = 0.03) and increased αSMA/MAC3 ratio (1.6 ± 0.3 vs. 0.7 ± 0.1, *p* = 0.01), which was also observed in proximal aortic plaques. Interestingly, a significant increase in TUNEL positive cells was observed in brachiocephalic artery plaques from *ApoE^−/−^ Gsdmd^−/−^* mice (141 ± 25 vs. 62 ± 8 cells/mm^2^, *p* = 0.005), indicating a switch to apoptosis. This switch from pyroptosis to apoptosis was also observed in vitro in *Gsdmd^−/−^* macrophages. In conclusion, targeting GSDMD appears to be a promising approach for limiting the transition to an inflammatory, vulnerable plaque phenotype.

## 1. Introduction

Vulnerable atherosclerotic plaques are characterized by a large necrotic core formed by excessive necrotic cell death and inflammation [1]. Plaque cells can undergo different types of regulated necrosis although their significance in atherosclerosis is not always clear-cut. One of the best-defined forms of regulated necrosis is pyroptosis, a pro-inflammatory form of regulated cell death that is characterized by the formation of plasma membrane pores via members of the gasdermin (GSDM) protein family [2,3]. Six members of this family have been identified, including gasdermin D (GSDMD). GSDMD is N-terminally (NT) cleaved and is activated by caspase 1 and caspase 4/5 (homologous to caspase 11 in mouse) [3,4,5]. Subsequently, NT-GSDMDs oligomerize, translocate to the cell membrane and induce pore formation, which allows the release of cellular content and pro-inflammatory cytokines such as IL-1β and IL-18, and finally results in membrane disruption and cell lysis [6,7].

In canonical pyroptosis induction, cleavage and activation of caspase 1 is mediated by inflammasomes, which are large supramolecular complexes that sense intracellular danger signals through nucleotide-binding oligomerization (NOD)-like receptor sensor molecules [8]. NLRP3 (NOD-like, leucine-rich repeat (LRR) and pyrin domain containing receptor 3) inflammasome-mediated pyroptosis is currently the best characterized pathway described in atherosclerotic plaques. Activation of the NLRP3 inflammasome can be initiated by oxidized LDL, cholesterol and calcium phosphate crystals inducing lysosomal lysis, cathepsin release, and potassium efflux [9,10,11,12]. In human atherosclerotic plaques, mRNA and protein levels of caspase 1, NLRP3, IL-1β, and IL-18 are increased compared to normal arteries [13,14,15,16]. Immunohistochemical analyses of human carotid plaques have revealed that NLRP3 immunoreactivity colocalizes with CD68-positive macrophages and occasionally with smooth muscle cells [15,16], and that plaque rupture is associated with strong immunoreactivity for caspase 1 [17]. In mice, several studies have demonstrated beneficial effects on atherogenesis and plaque stability when components of the NLRP3 inflammasome, caspase 1 activity, IL-1 or IL-18 are lacking [18,19,20,21,22,23,24,25,26]. Together, these studies suggest that modulation of NLRP3- and caspase 1-mediated pyroptosis offers significant health benefits for patients with advanced atherosclerosis [27]. This assumption is supported by the CANTOS trial showing beneficial effects of IL-1β antibody canakinumab in statin-treated patients at risk [28]. In contrast to canonical pyroptosis, caspase 4/5 and caspase 11 can be directly activated without the need for an inflammasome, resulting in non-canonical pyroptosis induction [29,30]. Recently, caspase 11 deficiency was reported to reduce plaque burden and macrophage infiltration in *ApoE^−/−^* mice [30], demonstrating that also non-canonical pyroptosis plays a role in atherogenesis.

GSDMD is the common executor of both canonical pyroptosis (mediated by NLRP3 and other inflammasomes) and non-canonical pyroptosis. Moreover, GSDMD is required for IL-1β release, not only during pyroptosis but also in viable macrophages [7,31]. Interestingly, *Gsdmd* mRNA is upregulated in peripheral blood monocytes from patients with coronary artery disease and expression of GSDMD and NT-GSDMD is increased in *ApoE^−/−^* mice fed a high fat diet as compared to chow-fed controls [30]. These experiments indicate that GSDMD is actively involved in pyroptosis during atherogenesis in both humans and mice, and thus represents a promising target in plaques for inhibiting pyroptosis and inflammation. Genetic deletion of *Gsdmd* or pharmacological inhibition with necrosulfonamide reduces infarct size and heart failure in a mouse model of acute myocardial infarction [32], underlining the involvement of GSDMD in cardiovascular disease and the possibility for using it as a pharmacological target in atherosclerosis. Therefore, we aimed to evaluate the impact of *Gsdmd* gene deletion in atherogenesis. First, the effects of *Gsdmd* gene deletion were evaluated in macrophages and smooth muscle cells in vitro. We also analyzed the expression of cleaved GSDMD in human carotid lesions. Next, the effect of *Gsdmd* deletion on advanced atherogenesis was evaluated in *ApoE^−/−^* mice.

## 2. Materials and Methods

### 2.1. Human Atherosclerotic Plaques

Human carotid endarterectomy specimens were obtained from patients (71 ± 3 years, 70% men) with a carotid stenosis of >70% [33]. Specimens were fixed in 4% formaldehyde (pH 7.4) within 2 min after surgical removal and paraffin embedded. To identify specific cell types expressing cleaved GSDMD, double immunohistochemical staining was performed using anti-cleaved GSDMD (Cell signaling, 36425, Danvers, MA, USA) combined with anti-CD68 (clone PG-M1, ab9498, Abcam, Cambridge, UK), anti-α-smooth muscle actin (αSMA; clone 1A4, A2547, Sigma-Aldrich, St. Louis, MO, USA), or anti-CD31 (clone JC/70A, ab783, Abcam, Cambridge, UK). Images were acquired with an Olympus BX43 microscope and quantified using Image J software (National Institutes of Health, Bethesda, MD, USA).

### 2.2. Mice

Standard *ApoE^−/−^* mice (Jackson Laboratory, 002052, Bar Harbor, ME, USA) were crossbred with *Gsdmd^−/−^* mice (Genentech, South San Francisco, CA, USA), carrying a 1 bp insertion in the *Gsdmd* coding sequence (GAGTGATGTTGTCAGGCATGGGA becomes GAGTGATGTT**t**GTCAGGCATGGGA) created with CRISPR/Cas9. Litters were screened for the *Gsdmd^−/−^* genotype by PCR analysis using Gsdmd-specific primers (forward primer: GTTTCTTGTCGATGGGAACATTCAG, reverse primer: TGAGTCACACGCAGTATA) followed by Sanger sequencing using the reverse primer. Genotyping of the *ApoE* alleles was performed by PCR according to the instructions from the Jackson Laboratory. Thereafter, *ApoE^−/−^ Gsdmd^−/−^* mice and *ApoE^−/−^ Gsdmd^+/+^* controls (all females, 6–8 weeks old) were fed a western-type diet (WD; TD.88137 supplemented with 21% fat and 0.2% cholesterol, Envigo, Indianapolis, IN, USA) to induce plaque formation. The animals were housed in a temperature-controlled room with a 12 h light/dark cycle and had free access to water and food. After 16 weeks WD, an overdose of sodium pentobarbital (250 mg/kg, i.p.) was administered and blood samples were collected via the retro-orbital plexus. Plasma levels of total cholesterol were measured using a commercially available kit (Randox laboratories, Crumlin, UK). Blood leukocyte subsets were analyzed on a BD accuri C6 flow cytometer as previously described [34]. All experiments were approved by the Ethical Committee of the University of Antwerp (Code 2019-24) and carried out in accordance with European Directive 2010/63/EEC.

### 2.3. Histological Analyses

The thoracic aorta was stained en face with Oil Red O to determine lipid burden. The heart, brachiocephalic artery, and proximal ascending aorta of *ApoE^−/−^ Gsdmd^−/−^* and *ApoE^−/−^ Gsdmd^+/+^* mice were fixed in 4% formaldehyde (pH 7.4) for 24 h, dehydrated overnight in 60% isopropanol, and subsequently embedded in paraffin. The proximal ascending aorta was marked on the distal arch end and the brachiocephalic artery on the distal carotid end to ensure that they were always cut on the proximal side. Serial cross-sections (5 µm) of the proximal parts of the brachiocephalic artery, proximal aorta and aortic root were prepared in random for histological analyses. Atherosclerotic plaque size and necrotic core area (defined as acellular areas with a threshold of 3000 µm^2^) were analyzed on hematoxylin-eosin (H&E) stained sections. Total collagen content was measured on Sirius red stained sections. Apoptosis was analyzed using an ApopTag Plus Peroxidase In Situ Apoptosis kit (Millipore, S7101, Burlington, VT, USA). For immunohistochemistry, the following antibodies were used: anti-MAC3 (BD Pharmingen, 550292, San Diego, CA, USA) and anti-α-smooth muscle actin (αSMA, 12547, Sigma-Aldrich, St. Louis, MO, USA). Images were acquired with an Olympus BX43 microscope, which was calibrated for each magnification. Plaque size was measured based on pixels per µm, which was determined during the calibration of the microscope. Per mouse, one section was analyzed. Plaques were manually delineated in Image J software (National Institutes of Health) to establish the region of interest (ROI). Further analyses within the ROIs were performed using color thresholding or manual counting (apoptotic cells).

### 2.4. Cell Culture

Bone marrow-derived macrophages (BMDMs) were harvested by flushing bone marrow of the femur with a 25 Gauge needle and heparinized (10 IU/mL) RPMI 1640 medium (Gibco Life Technology, Merelbeke, Belgium). After washing and filtration, cells were cultured in RPMI 1640 medium supplemented with Glutamax (Gibco Life Technology, Merelbeke, Belgium) and 15% L929-cell conditioned medium (LCCM) containing monocyte colony stimulating factor (M-CSF) for 7 days in 95% air/5% CO_2_ until 80–90% confluency was reached. To induce pyroptosis, BMDMs were primed with 100 ng/mL lipopolysaccharide (LPS, Sigma-Aldrich, St. Louis, MO, USA) for 4 h followed by treatment with 2.5–20 μM nigericin (Enzo Life Sciences, BML-CA421-0005, Brussels, Belgium) or 5 mM ATP (Calbiochem, 1191, Sigma-Aldrich, St. Louis, MO, USA). Vascular smooth muscle cells (VSMCs) were isolated as previously described [35,36]. Briefly, aortas were incubated in an enzyme solution containing 1 mg/mL collagenase type II (Worthington, Lakewood, NJ, USA), 1 mg/mL soybean trypsin inhibitor (Worthington, Lakewood, NJ, USA), and 0.744 units/mL elastase (Worthington) for 15 min at 37 °C to remove the adventitia. Subsequently, aortas were placed in a fresh enzyme solution for 75 min at 37 °C. Isolated cells were collected, washed, and resuspended in DMEM/F12 medium (Gibco Life Technology) supplemented with 20% heat-inactivated FBS (Gibco Life Technology). Cells were used from passage 4 till 10 and cultured in DMEM/F12 medium supplemented with 10% heat-inactivated FBS. To induce pyroptosis, VSMCs were primed with 50 ng/mL TNFα (Abcam, ab9740, Cambridge, UK) for 2 h followed by treatment with 5 mM ATP (Calbiochem, 1191) for 2 h. Alternatively, non-primed cells were treated with 300 µg/mL oxidized LDL (oxLDL, L34357, Life Technologies, Carlsbad, CA, USA) for 48 h. Necrosis was evaluated by labeling BMDMs or VSMCs with 1 µg/mL propidium iodide (PI, Molecular Probes, Eugene, OR, USA) and 10 µg/mL Hoechst (Life Technologies, Carlsbad, CA, USA), followed by visualization of PI/Hoechst-labeled cells using a Celena S digital Imaging System (Logos Biosystems, Dongan-gu, Anyang-si, Korea). To measure the release of IL-1β and IL-18, primed BMDMs were treated with 10 μM nigericin. After 2 h, cell supernatant was collected and cellular debris was removed by centrifugation. IL-1β and IL-18 secretions were quantified in the supernatant using a mouse IL-1β Quantikine ELISA kit (R&D Systems, MLB00C, Minneapolis, MN, USA) and IL-18 mouse ELISA kit (Invitrogen, BMS618-3, Waltham, MA, USA), respectively.

### 2.5. Flow Cytometry

Apoptosis was quantified with TdT-mediated dUTP-X nick end labeling (TUNEL). Briefly, BMDMs were detached with 0.25% trypsin-EDTA (Thermo Fischer Scientific, 25200072, Waltham, MA, USA). Detached BMDMs were fixed in 4% paraformaldehyde for 1 h at room temperature. After washing with PBS, BMDMs were permeabilized with 0.1% Triton X-100 in 0.1% sodium citrate solution for 2 min on ice. BMDMs were washed again and incubated with TUNEL reaction mixture using an in situ cell death detection kit (fluorescein, 11684795910, Roche, Switzerland) for 1 h at 37 °C. The samples were washed twice with FACS buffer (PBS with 0.1% bovine serum albumin and 0.05% sodium azide) and measured in the FL-1 channel on a BD Accuri C6 flow cytometer. At least 10,000 cells were measured. Debris was always gated out based on FSC/SSC scatter. Positive controls consisted of BMDMs treated with TNFα combined with cycloheximide. Negative controls consisted of untreated BMDMs. Unstained controls were included to exclude background signal.

### 2.6. Western Blotting

Tissues were homogenized in RIPA buffer containing protease and phosphatase inhibitors. Protein concentrations were determined using the BCA method. Samples were then 1:1 diluted in Laemmli sample buffer (Bio-Rad, Hercules, CA, USA) containing 5% β-mercaptoethanol (Sigma-Aldrich) and heat-denatured for 5 min at 100 °C. Samples were loaded on Bolt 4–12% Bis-Tris gels (Invitrogen) and after electrophoresis transferred to Immobilon-FL PVDF membranes (Millipore) according to standard procedures. Subsequently, membranes were blocked for one hour in Odyssey Li-COR blocking buffer. After blocking, membranes were probed with primary antibodies diluted in Odyssey Li-COR blocking buffer followed by 1 h incubation with IRDye-labeled secondary antibodies at room temperature. Membranes were visualized with an Odyssey SA infrared imaging system (Li-COR Biosciences, Lincoln, NE, USA).

The following primary antibodies were used: rabbit anti-GSDMD (Abcam, ab219800, Cambridge, UK), rabbit anti-cleaved GSDMD (Cell signaling, 10137, Danvers, MA, USA), rabbit anti-NLRP3 (Abcam, ab263899), rabbit anti-caspase 1 (Abcam, ab179515), and mouse anti-β-actin (Abcam, ab8226).

### 2.7. Statistical Analyses

Statistical analyses were performed using Graphpad Prism 9. All data were expressed as mean ± SEM, except in boxplots where medians are shown. Dots represent the number of samples from independent experiments or individual mice. Statistical tests are specified in the text and figure legends. Differences were considered significant when *p* < 0.05.

## 3. Results

### 3.1. Pyroptosis Is Inhibited in Gsdmd^−/−^ BMDMs but a Switch to Apoptosis Is Induced

**Figure 1:**
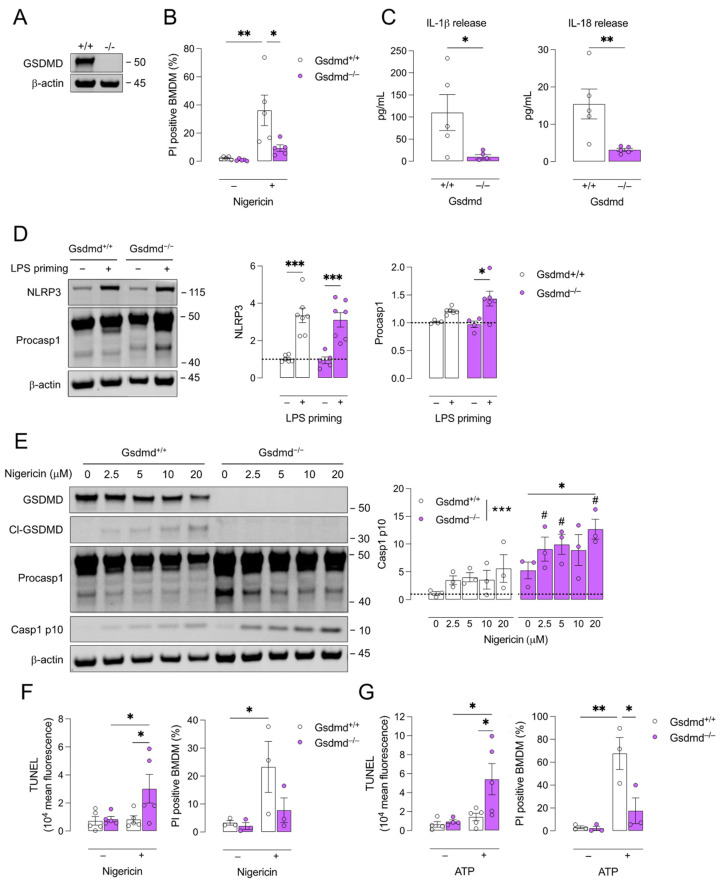
Pyroptosis is inhibited in *Gsdmd^−/−^* BMDMs and a switch to apoptosis occurs. Bone marrow-derived macrophages (BMDMs) were isolated from *Gsdmd^+/+^* and *Gsdmd^−/−^* mice. (**A**) Deficiency of GSDMD in *Gsdmd^−/−^* BMDMs was confirmed via Western blotting. (**B**–**D**) *Gsdmd^−/−^* and *Gsdmd^+/+^* BMDMs were primed with 100 ng/mL LPS for 4 h followed by treatment with 10 μM nigericin for 2 h. (**B**) Cell death was measured using propidium iodide (PI) labelling (two-way ANOVA followed by Sidak’s multiple comparison, *n* = 5 independent experiments) and (**C**) the release of IL-1β and IL-18 was quantified in the cell supernatant (Mann–Whitney test, *n* = 5 independent experiments). (**D**) Western blot analyses of NLRP3 and procaspase (procasp) 1 on lysates of LPS-primed BMDMs (two-way ANOVA followed by Sidak’s multiple comparison, *n* = 4–7 independent experiments, data are expressed as fold change of target/β-actin ratio). (**E**) *Gsdmd^−/−^* and *Gsdmd^+/+^* BMDMs were primed with 100 ng/mL LPS for 4 h followed by treatment with 2.5–20 μM nigericin for 2 h. Western blot analyses of GSDMD, cleaved (cl)-GSDMD, procaspase 1, and caspase (casp) 1 p10 (two-way ANOVA followed by Dunnett’s multiple comparison between concentrations per genotype, * p < 0.05, *** p < 0.001; two-way ANOVA followed by Sidak’s multiple comparison between genotypes per concentration, *# p* < 0.05; *n* = 3 independent experiments, data are expressed as fold change of target/β-actin ratio). (**F**,**G**) LPS-primed BMDMs were treated for 1 h with (**F**) 20 μM nigericin or (**G**) 5 mM ATP. TUNEL labeling was performed and analyzed via flow cytometry and cell death was measured using PI labelling (two-way ANOVA followed by Sidak’s multiple comparison, *n* = 3–5 independent experiments). * *p* < 0.05, ** *p* < 0.01, *** *p* < 0.001.

Because pyroptosis is mainly characterized in myeloid cells [37,38], BMDMs were isolated from *Gsdmd^−/−^* and *Gsdmd^+/+^* mice. First, GSDMD deficiency was confirmed in *Gsdmd^−/−^* BMDMs via Western blotting (Figure 1A). To induce pyroptosis, BMDMs were primed with LPS followed by treatment with nigericin, a canonical pyroptosis inducer. Significant induction of cell death was observed in *Gsdmd^+/+^* cells while *Gsdmd^−/−^* cells were clearly more resistant to pyroptosis induction (Figure 1B). The release of IL-1β and IL-18 was quantified in BMDM supernatant after LPS priming and treatment with nigericin. Both IL-1β and IL-18 were released by *Gsdmd^+/+^* BMDMs and this was significantly decreased in *Gsdmd^−/−^* BMDMs (Figure 1C). IL-1β and IL-18 were not detected in supernatant of untreated BMDMs (data not shown). LPS priming induced the expression of NLRP3 and procaspase 1, both in *Gsdmd^+/+^* and *Gsdmd^−/−^* BMDMs (Figure 1D). Accordingly, Western blot analysis further showed that LPS-primed *Gsdmd^+/+^* BMDMs treated with 2.5 to 20 μM nigericin expressed GSDMD and cleaved GSDMD, while *Gsdmd^−/−^* BMDMs did not (Figure 1E). Moreover, both *Gsdmd^+/+^* and *Gsdmd^−/−^* BMDMs expressed procaspase 1 and, upon treatment with nigericin, the active caspase 1 p10 subunit was expressed (Figure 1E). Altogether, these results confirm that GSDMD-pore formation and pyroptosis induction are inhibited in *Gsdmd^−/−^* BMDMs while upstream NLRP3 assembly and procaspase 1 recruitment (and cleavage) are not affected.

Interestingly, the expression of caspase 1 p10 was significantly higher in *Gsdmd^−/−^* BMDMs as compared to *Gsdmd^+/+^* controls (Figure 1E). Because active caspase 1 can also act in a pro-apoptotic fashion [39,40], a TUNEL assay was performed on LPS-primed BMDMs after treatment with nigericin for 1 h (Figure 1F). A significant increase in TUNEL positivity was observed after nigericin treatment in *Gsdmd^−/−^* BMDMs, while PI positivity was not increased, indicating that the plasma membranes of *Gsdmd^−/−^* cells were intact but that DNA fragmentation typical of apoptosis occurred. In contrast, TUNEL positivity did not change in *Gsdmd^+/+^* BMDMs after nigericin treatment while PI positivity did increase significantly (Figure 1F). Similar findings were observed after treatment with ATP, another classical caspase 1-dependent pyroptosis inducer (Figure 1G).

### 3.2. Gsdmd^−/−^ VSMCs Are Less Sensitive to Pyroptosis Inducers

**Figure 2:**
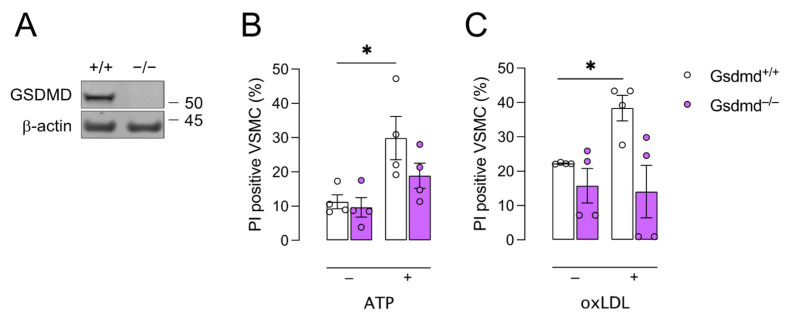
*Gsdmd^−/−^* VSMCs are less sensitive to pyroptosis inducers compared to controls. Vascular smooth muscle cells (VSMCs) were isolated from *Gsdmd^+/+^* and *Gsdmd^−/−^* mice. (**A**) Deficiency of GSDMD in *Gsdmd^−/−^* VSMCs was confirmed via Western blotting. (**B**) VSMCs were primed with 50 ng/mL TNFα for 2 h followed by treatment with 5mM ATP for 1 h. (**C**) VSMCs were treated with 300 μg/mL oxLDL for 48 h. Cell death was measured using PI labelling. * *p* < 0.05 (two-way ANOVA followed by Sidak’s multiple comparison, *n* = 4 independent experiments).

There is increasing evidence that pyroptosis is not limited to inflammatory cells [22,41,42,43]. Therefore, VSMCs were isolated from *Gsdmd^+/^*^+^ and *Gsdmd^−/−^* mice. Absence of GSDMD in *Gsdmd^−/−^* VSMCs was confirmed via Western blotting (Figure 2A). To induce pyroptosis, VSMCs were primed with TNFα followed by ATP treatment. Cell death increased significantly in *Gsdmd^+/+^* VSMCs but not in *Gsdmd^−/−^* VSMCs (Figure 2B). Similar to TNFα/ATP treatment, oxLDL, which is also reported to induce pyroptosis [12,44], increased cell death significantly in *Gsdmd^+/+^* VSMCs but not in *Gsdmd^−/−^* cells (Figure 2C).

### 3.3. Cleaved GSDMD Is Expressed in Human Carotid Lesions

**Figure 3:**
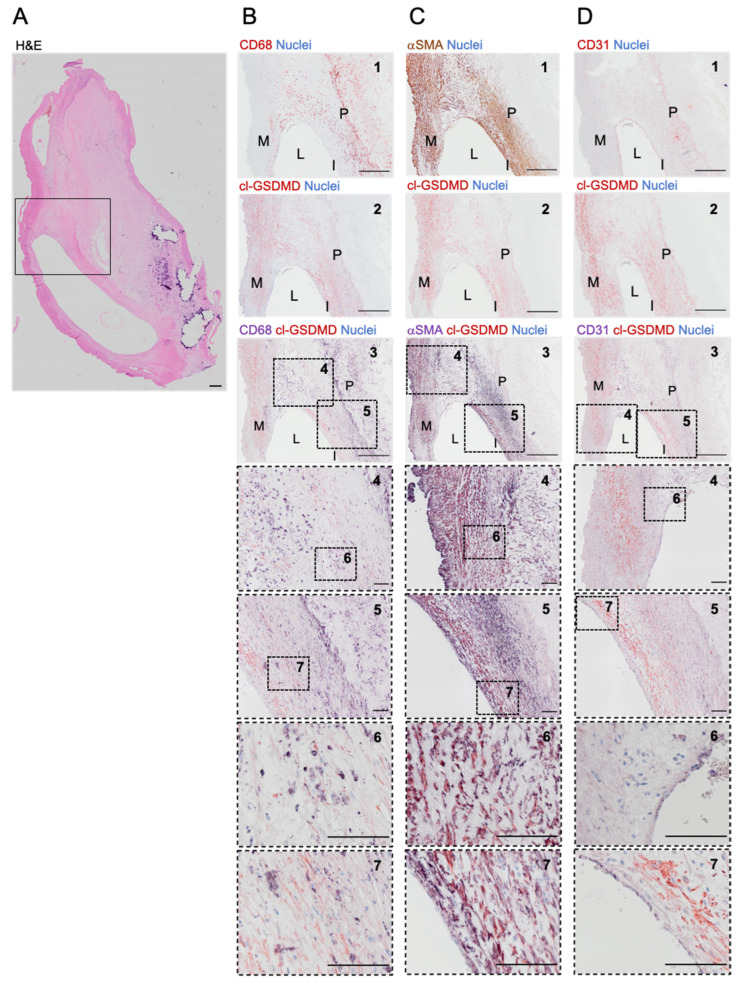
Cleaved GSDMD is expressed in human carotid plaques. (**A**) Overview image of a section from a human carotid artery lesion stained with hematoxylin/eosin. The boxed area corresponds with the region shown in (**B**–**D**). (**B**–**D**) Immunohistochemical staining of cleaved (cl)-GSDMD combined with (**B**) CD68, (**C**) α-smooth muscle actin (αSMA) or (**D**) CD31. From top to bottom: 1. Image of section stained for (B) CD68 (red), (**C**) αSMA (brown), or (**D**) CD31 (red). 2. Image of section stained for cl-GSDMD (red). 3. Image of double stained section for cl-GSDMD (red) combined with (**B**) CD68, (**C**) αSMA, or (**D**) CD31 (purple). 4–7. Magnifications of dotted frames in images 3–5. Scale bar = 500 μm (**A**,**B**–**D**: 1–3), 100 μm (**B**–**D**: 4–7). Representative images are shown. M = media, L = lumen, I = intima, P = plaque.

To confirm that GSDMD is not only active in mice, cell-specific expression of cleaved GSDMD was evaluated in human carotid lesions (Figure 3). In general, cleaved GSDMD was expressed in the plaque, especially in the shoulder regions, and media (Figure 3B–D). Double immunohistochemical staining of cleaved GSDMD and CD68 showed that GSDMD is cleaved in plaque areas rich in macrophages, albeit not all CD68-positive cells contained cleaved GSDMD (Figure 3B). On sections stained separately for cleaved GSDMD and αSMA (Figure 3C), the positivity pattern appeared very similar. Indeed, double immunohistochemical staining of cleaved GSDMD and αSMA showed a clear overlapping of red (cl-GSDMD) and purple (αSMA) signal, confirming colocalization of cleaved GSDMD and smooth muscle cells. The CD31-positive intima delineated the vessel lumen (Figure 3D). However, no cleaved GSDMD positivity was observed in CD31-positive endothelial cells.

### 3.4. Atherogenesis Is Delayed in ApoE^−/−^ Gsdmd^−/−^ Mice but a Switch to Apoptosis Occurs in Plaques of the Brachiocephalic Artery

**Figure 4:**
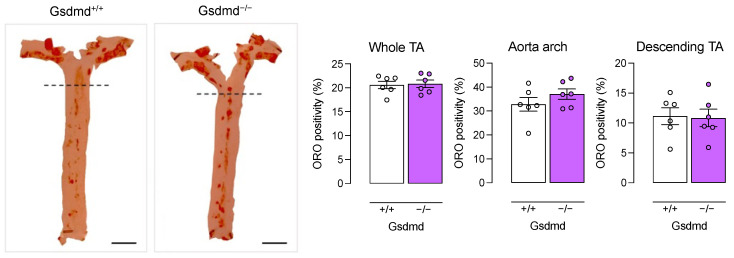
Lipid burden in the thoracic aorta is not different between *ApoE^−/−^ Gsdmd^−/−^* and *ApoE^−/−^ Gsdmd^+/+^* mice. *ApoE^−/−^ Gsdmd^−/−^* and *ApoE^−/−^ Gsdmd^+/+^* mice were fed a WD for 16 weeks. The aortic arch and descending thoracic aorta (TA) were stained en face with Oil Red O to evaluate the plaque burden (Mann–Whitney test, *n* = 6 mice per group). The dotted line seperates the aortic arch (top) from the descending TA (bottom). Scale bar = 5 mm.

To evaluate the effect of *Gsdmd* gene deletion on atherogenesis, *ApoE^−/−^ Gsdmd^−/−^* and *ApoE^−/−^ Gsdmd^+/+^* mice were fed a western-type diet (WD) for 16 weeks. First, deletion of *Gsdmd* was confirmed via Western blotting in the aortic arch, descending thoracic aorta (desc TA), lung, liver, heart, spleen, and kidney of *ApoE^−/−^ Gsdmd^−/−^* mice (Supplementary Figure S1). After 16 weeks WD, body weight (27.2 ± 0.7 vs. 29.3 ± 1.0 g, 1.5 ± 0.03 vs. 1.5 ± 0.05 fold change compared to starting weight; independent samples *t*-test, *p* > 0.05), plasma cholesterol (685.8 ± 18.2 vs. 652.1 ± 20.0 mg/dL; independent samples *t*-test, *p* > 0.05), and circulating leukocyte subsets (Supplementary Figure S2) were not different between *ApoE^−/−^ Gsdmd^+/+^* and *ApoE^−/−^ Gsdmd^−/−^* mice. En face Oil Red O staining of the thoracic aorta showed no differences in lipid burden between *ApoE^−/−^ Gsdmd^+/+^* and *ApoE^−/−^ Gsdmd^−/−^* mice in the whole thoracic aorta, as well as in the aortic arch and descending aorta separately (Figure 4).

**Figure 5:**
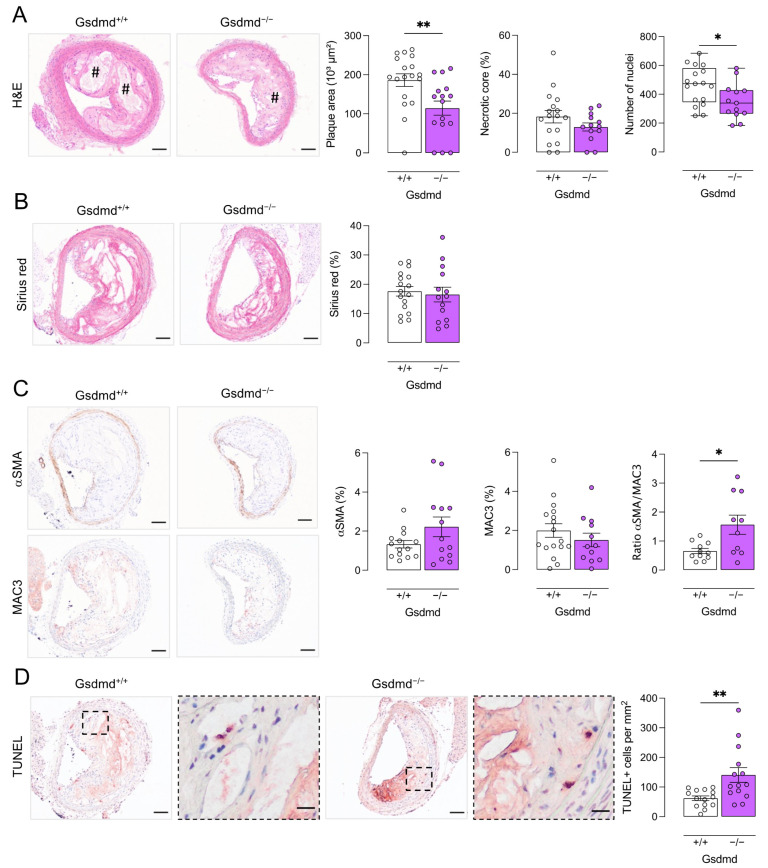
Plaques in the brachiocephalic artery from *ApoE^−/−^ Gsdmd^−/−^* mice are smaller but show increased apoptosis. *ApoE^−/−^ Gsdmd^−/−^* and *ApoE^−/−^ Gsdmd^+/+^* mice were fed a WD for 16 weeks. Sections of the brachiocephalic artery were stained with (**A**) hematoxylin/eosin to quantify plaque size, necrotic cores (# hash signs), and cell infiltration; (**B**) Sirius red to measure total collagen content; (**C**) anti-MAC3 and anti-α-smooth muscle actin (αSMA) to determine macrophage and smooth muscle cell content, respectively, and to calculate the ratio of αSMA/MAC3 immunoreactivity; (**D**) TUNEL to count apoptotic cells (dotted boxes are magnified, scale bar = 20 μm). * *p* < 0.05, ** *p* < 0.01 (independent samples *t*-test, boxplot: Mann–Whitney test, *n* = 10–18 mice per group). Scale bar = 100 μm. Representative images are shown.

In contrast, plaque size was significantly decreased in the brachiocephalic artery of *ApoE^−/−^ Gsdmd^−/−^* mice as compared to *ApoE^−/−^ Gsdmd^+/+^* controls (115 ± 18 vs. 186 ± 16 × 10^3^ µm^2^; independent samples *t*-test, *p* = 0.006, Figure 5A). This suggests that plaque progression is delayed in *ApoE^−/−^ Gsdmd^−/−^* mice. Moreover, the necrotic core area was significantly decreased in *ApoE^−/−^ Gsdmd^−/−^* plaques of the brachiocephalic artery as compared to *ApoE^−/−^ Gsdmd^+/+^* controls (19 ± 4 vs. 37 ± 7 × 10^3^ µm^2^; independent samples *t*-test, *p* = 0.03), although the relative necrotic core area was not altered (Figure 5A). Similarly, the number of cells infiltrated in plaques of the brachiocephalic artery of *ApoE^−/−^ Gsdmd^−/−^* mice was significantly lower as compared to *ApoE^−/−^ Gsdmd^+/+^* mice (Figure 5A). The total collagen content did not differ between *ApoE^−/−^ Gsdmd^−/−^* and *ApoE^−/−^ Gsdmd^+/+^* plaques in the brachiocephalic artery (Figure 5B). Moreover, αSMA and MAC3 immunoreactivity did not change significantly, however, the ratio of αSMA to MAC3 immunoreactivity increased significantly in *ApoE^−/−^ Gsdmd^−/−^* plaques of the brachiocephalic artery as compared to controls (Figure 5C), indicating a shift in plaque composition and inflammatory state. Because a switch in cell death modality was observed in vitro in *Gsdmd^−/−^* BMDMs, TUNEL staining was performed on plaques *of ApoE^−/−^ Gsdmd^+/+^* and *ApoE^−/−^ Gsdmd^−/−^* mice. Similarly to what was observed in vitro, TUNEL positivity in vivo was significantly increased in plaques of the brachiocephalic artery from *ApoE^−/−^ Gsdmd^−/−^* mice compared to *ApoE^−/−^ Gsdmd^+/+^* controls (Figure 5D).

**Figure 6:**
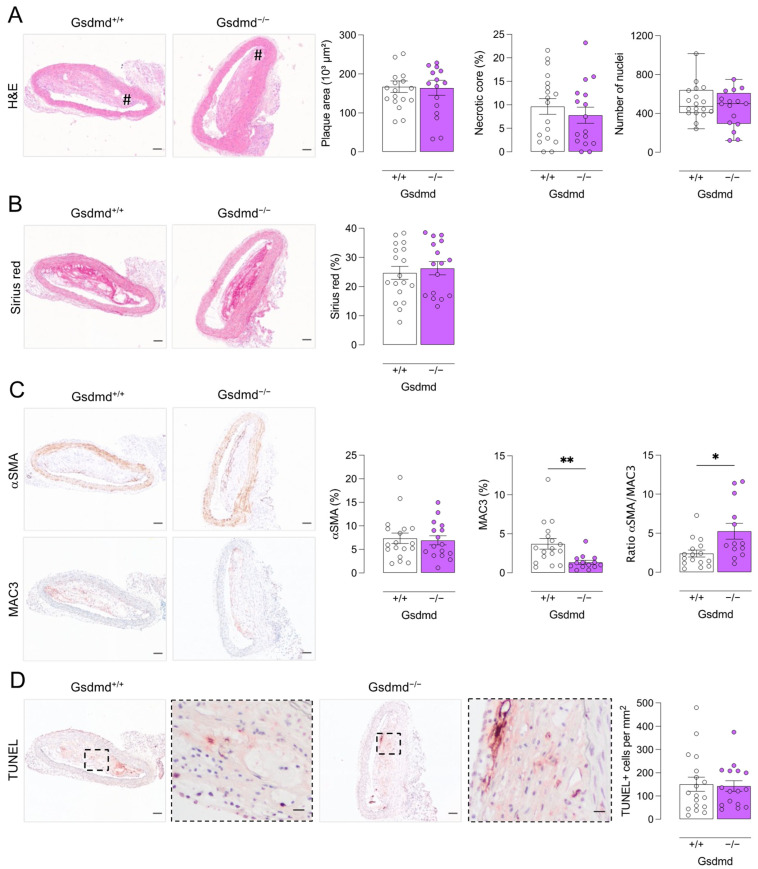
Plaques in the proximal aorta from *ApoE^−/−^ Gsdmd^−/−^* mice show decreased macrophage infiltration as compared to *ApoE^−/−^ Gsdmd^+/+^* controls. *ApoE^−/−^ Gsdmd^−/−^* and *ApoE^−/−^ Gsdmd^+/+^* mice were fed a WD for 16 weeks. Sections of the proximal aorta were stained with (**A**) hematoxylin/eosin to quantify plaque size, necrotic cores (# hash signs), and cell infiltration; (**B**) Sirius red to measure total collagen content; (**C**) anti-MAC3 and anti-α-smooth muscle actin (αSMA) to determine macrophage and smooth muscle cell content, respectively, and to calculate the ratio of αSMA/MAC3 immunoreactivity; (**D**) TUNEL to count apoptotic cells (dotted boxes are magnified, scale bar = 20 μm). * *p* < 0.05, ** *p* < 0.01 (independent samples *t*-test, boxplot: Mann–Whitney test, *n* = 13–18 mice per group). Scale bar = 100 μm. Representative images are shown.

In plaques of the proximal aorta (Figure 6) and aortic root (Supplementary Figure S3), no differences in plaque size, necrotic core area, cell infiltration, and total collagen content were observed between *ApoE^−/−^ Gsdmd^−/−^* and *ApoE^−/−^ Gsdmd^+/+^* mice. Nevertheless, MAC3 immunoreactivity was significantly decreased while the αSMA to MAC3 immunoreactivity ratio was significantly increased in plaques of the proximal aorta from *ApoE^−/−^ Gsdmd^−/−^* mice, again indicating a shift in plaque composition and inflammatory state (Figure 6C). In contrast to what was observed in vitro and in the brachiocephalic artery from *ApoE^−/−^ Gsdmd^−/−^* mice, TUNEL positivity was not increased in plaques in the proximal aorta (Figure 6D).

## 4. Discussion

GSDMD is the common executor of canonical and non-canonical pyroptosis. Increasing evidence suggests that pyroptosis occurs in atherosclerotic plaques in both humans and mice [13,14,15,16,17,18,19,20,21,22,23,24,25,26]. In the present study, we isolated BMDMs and VSMCs from *Gsdmd^+/+^* and *Gsdmd^−/−^* mice and confirmed that canonical pyroptosis is GSDMD-dependent in BMDMs and VSMCs. Indeed, treatment of LPS-primed BMDMs with classical caspase 1 activators such as nigericin and ATP induced significant pyroptosis in *Gsdmd^+/+^* controls, but not in *Gsdmd^−/−^* cells. Concurrently, *Gsdmd^−/−^* BMDMs showed increased DNA fragmentation, measured with TUNEL staining, which was absent in *Gsdmd^+/+^* BMDMs. Thus, *Gsdmd^−/−^* BMDMs are resistant to canonical pyroptosis induction, albeit they switch to another type of cell death that is characterized by DNA fragmentation, strongly suggestive of apoptosis. A switch from pyroptosis to apoptosis is further supported by the observation that caspase 1 p10 is upregulated in LPS/nigericin-treated *Gsdmd^−/−^* BMDMs, and not in *Gsdmd^+/+^* controls, and may act in a pro-apoptotic manner in the absence of GSDMD [39,40,45].

Previous studies have demonstrated that GSDMD, NT-GSDMD, and *Gsdmd* mRNA are upregulated in aortas of hyperlipidemic mice and in LPS/oxLDL-treated mouse peritoneal macrophages [30]. Moreover, the expression of NT-GSDMD is increased in plaques of LDLr^−/−^ mice [45]. *Gsdmd* mRNA is also upregulated in peripheral blood monocytes of atherosclerotic patients [30]. However, to the best of our knowledge, the expression of cleaved NT-GSDMD has not yet been evaluated in human atherosclerotic plaques. Therefore, we performed immunostaining on human carotid lesions. We demonstrated that NT-GSDMD was expressed in human plaques, both in macrophage- and VSMC-rich regions. However, it should be noted that this finding was based on analysis of a limited number of plaques and comparison with plaque-free arteries was not made. Other groups have shown that components of the canonical pyroptosis pathway colocalize with plaque macrophages and contribute to macrophage pyroptosis and atherogenesis [12,15,24,27]. Interestingly, in the present study, colocalization of NT-GSDMD with αSMA-positive smooth muscle cells was even more pronounced than with CD68-positive macrophages. This is in line with a recent study reporting that caspase 1-dependent pyroptosis occurs in VSMCs and contributes to the progression of atherosclerosis [22]. Importantly, pyroptosis has also been described in plaque endothelial cells [46,47,48]. However, we did not observe any NT-GSDMD immunoreactivity in the luminal endothelial layer of human carotid lesions. Similarly, Rajamäki and colleagues also did not observe any NLRP3 immunoreactivity in the endothelium of human coronary plaques [15].

Disulfiram (used to treat alcohol use disorder) has recently been identified as a potent GSDMD inhibitor [49]. Furthermore, dimethyl fumarate (used as an immunomodulator in multiple sclerosis) also covalently binds GSDMD thereby inhibiting pyroptosis in vitro and in vivo in a mouse model of LPS-induced shock [50]. The availability of safe, EMA- and FDA-approved drugs that inhibit GSDMD makes it an interesting therapeutic target to address pyroptosis in atherosclerosis. Therefore, we crossbred atherosclerotic *ApoE^−/−^* mice with *Gsdmd^−/−^* mice to evaluate the effect of GSDMD deficiency on atherogenesis. After 16 weeks WD, Oil red O staining was performed on the thoracic aorta but no change in overall lipid burden was observed. In line with this finding, plaque size was not altered in the proximal aorta. However, the plaque size in the brachiocephalic artery of *ApoE^−/−^ Gsdmd^−/−^* mice was significantly decreased as compared to *ApoE^−/−^ Gsdmd^+/+^* mice. Although both the ascending proximal aorta and the brachiocephalic artery of *ApoE^−/−^* mice are atherogenesis-prone sites [51], plaques in the brachiocephalic artery enter an advanced, human-like stage more rapidly [52,53]. Indeed, plaques in the brachiocephalic artery of *ApoE^−/−^* mice easily reveal a vulnerable plaque phenotype and thinning of the fibrous cap may even lead to plaque rupture, similarly to human plaques [53,54,55]. In contrast, aortic plaques of *ApoE^−/−^* mice resemble stable lesions in humans, based on fibrous cap stress analysis [53]. Altogether, these observations suggest that GSDMD deficiency does not affect plaque initiation (as lipid burden in the thoracic aorta and proximal aortic plaque size were not decreased in *ApoE^−/−^ Gsdmd^−/−^* mice) but rather the transition to and growth of a vulnerable plaque. This is supported by the increased αSMA/MAC3-immunoreactivity ratio observed in plaques of both the brachiocephalic artery and the proximal aorta of *ApoE^−/−^ Gsdmd^−/−^* mice as compared to controls. This finding suggests a lower degree of plaque inflammation and vulnerability, and was also observed in plaques of the proximal aorta despite the unchanged plaque size, demonstrating a general decrease in plaque inflammation. Moreover, macrophage content was significantly decreased in plaques in the proximal aorta of *ApoE^−/−^ Gsdmd^−/−^* mice as compared to controls. This is in line with a recent study in which GSDMD expression was suppressed in *ApoE^−/−^* mice using an adeno-associated virus-5 (AAV-5) delivery system [30]. The authors reported a decrease in F4/80-positive macrophage content in the aorta and aortic valve of AAV-5-GSDMD-treated *ApoE^−/−^* mice as compared to AAV-5-control-treated *ApoE^−/−^* mice. Of note, the authors also reported a decrease in lipid burden and plaque size in the aorta and aortic valve when GSDMD expression was suppressed, however, only a limited number of mice was included and preliminary data were reported [30]. Another study reported a decreased lesion area in the aortic root of LDLr antisense oligonucleotide-treated *Gsdmd^−/−^* mice compared to *Gsdmd^+/+^* controls, which was attributed to decreased IL-1β release resulting in decreased foam cell formation and ATP release in plasma [45]. Importantly, experimental differences such as a longer period of feeding WD in the present study, evaluation of different vascular sites, and the use of different genetic models make comparison with these studies difficult.

In plaques of the brachiocephalic artery, the number of TUNEL positive cells per mm^2^ was significantly increased in *ApoE^−/−^ Gsdmd^−/−^* mice compared to controls. Increased TUNEL positivity is not specific and DNA fragmentation is reported to occur during apoptotic death as well as during different forms of necrotic death [56,57]. However, as necrosis is decreased in plaques of *ApoE^−/−^ Gsdmd^−/−^* mice, it is plausible to conclude that a switch to apoptosis occurs when pyroptosis is defective. Accordingly, in vitro TUNEL positivity and levels of pro-apoptotic caspase 1 p10 were increased in *Gsdmd^−/−^* BMDMs while PI positivity did not increase. Similarly, Opoku and colleagues recently reported increased apoptosis, characterized by phosphatidyl serine exposure on the cell surface, when pyroptosis was defective in *Gsdmd^−/−^* macrophages [45]. Importantly, a switch to apoptosis, which is non-lytic and non-inflammatory (in contrast to pyroptosis), will limit plaque progression, inflammation, and destabilization and thus, is regarded to be atheroprotective at this stage of atherosclerosis in *ApoE^−/−^* mice. However, as previously reported by our lab, accumulation of apoptotic bodies can induce secondary necrosis, eventually resulting in expansion of the necrotic core and plaque size during later stages of atherosclerosis [58]. Accordingly, large plaques with an inflammatory phenotype were observed in the aortic root from both *ApoE^−/−^ Gsdmd^−/−^* and *ApoE^−/−^ Gsdmd^+/+^* mice, possibly because after 16 weeks WD plaques in the aortic root are in a more advanced stage compared to plaques in the brachiocephalic artery. Thus, the effects in the longer term of targeting GSDMD and the concomitant switch in cell death modality remain to be elucidated.

## 5. Conclusions

We report that cleaved GSDMD is present in human atherosclerotic plaques and is required for inflammatory pyroptosis in murine macrophages and smooth muscle cells in vitro. GSDMD deficiency in *ApoE^−/−^* mice does not inhibit plaque initiation and the formation of stable aortic plaques, but plays a role in the formation of inflammatory plaques in the brachiocephalic artery. Indeed, a shift toward a less inflammatory plaque composition and delayed plaque progression were observed in plaques of the brachiocephalic artery from *ApoE^−/−^ Gsdmd^−/−^* mice as compared to plaques with a more vulnerable phenotype observed in *ApoE^−/−^ Gsdmd^+/+^* controls. This is accompanied by less plaque necrosis and a switch to apoptosis when GSDMD is deficient, which is also observed in vitro in BMDMs. Therefore, targeting GSDMD appears to be a promising approach for limiting the transition to an inflammatory and vulnerable plaque phenotype, and subsequently plaque destabilization. However, the pharmacological translation and therapeutic value in atherosclerosis patients should be further investigated.

## Data Availability

The data that support the findings of this study are available from the corresponding author upon reasonable request.

## Supplementary Materials

The following supporting information can be downloaded at: https://www.mdpi.com/article/10.3390/biomedicines10051171/s1, Figure S1: Validation of GSDMD deficiency in tissues from *ApoE^−/−^ Gsdmd^−/−^* mice; Figure S2: Analysis of leukocyte subsets in plasma from *ApoE^−/−^ Gsdmd^−/−^* and *ApoE^−/−^ Gsdmd^+/+^* mice. Figure S3: Plaque analysis in the aortic root from *ApoE^−/−^ Gsdmd^−/−^* and *ApoE^−/−^ Gsdmd^+/+^* mice.
