# Supplementary material for: Gasdermin D Deficiency Limits the Transition of Atherosclerotic Plaques to an Inflammatory Phenotype in ApoE Knock-Out Mice
Source: Biomedicines. 2022 May 19;10(5):1171. doi: 10.3390/biomedicines10051171 (PMC9138554; doi:10.3390/biomedicines10051171)
Supplement: Supplementary file 1 [file biomedicines-10-01171-s001.zip › Biomedicines Gsdmd supplementary figures.pdf]

Figure S1

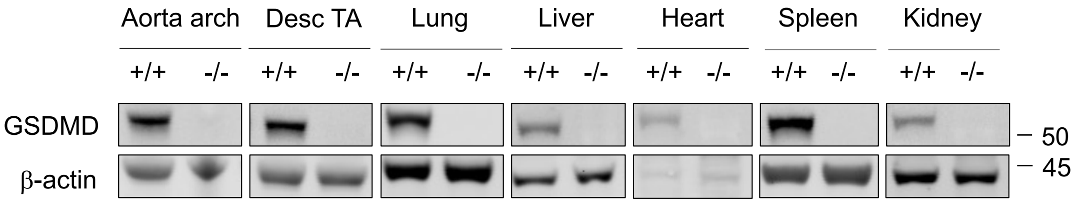

Validation of *Gsdmd* knock-out in tissue samples from *ApoE$^{-/-}$ Gsdmd$^{-/-}$* mice. *ApoE$^{-/-}$ Gsdmd$^{-/-}$* and *ApoE$^{-/-}$ Gsdmd$^{+/+}$* mice were fed a WD for 16 weeks. Lysates of the aorta arch, descending (desc) thoracic aorta (TA), lungs, liver, heart, spleen and kidney were prepared for western blot analyses of GSDMD expression. β-actin was used as a loading control. Representative blots are shown (n=10-18 mice per group).

Figure S2

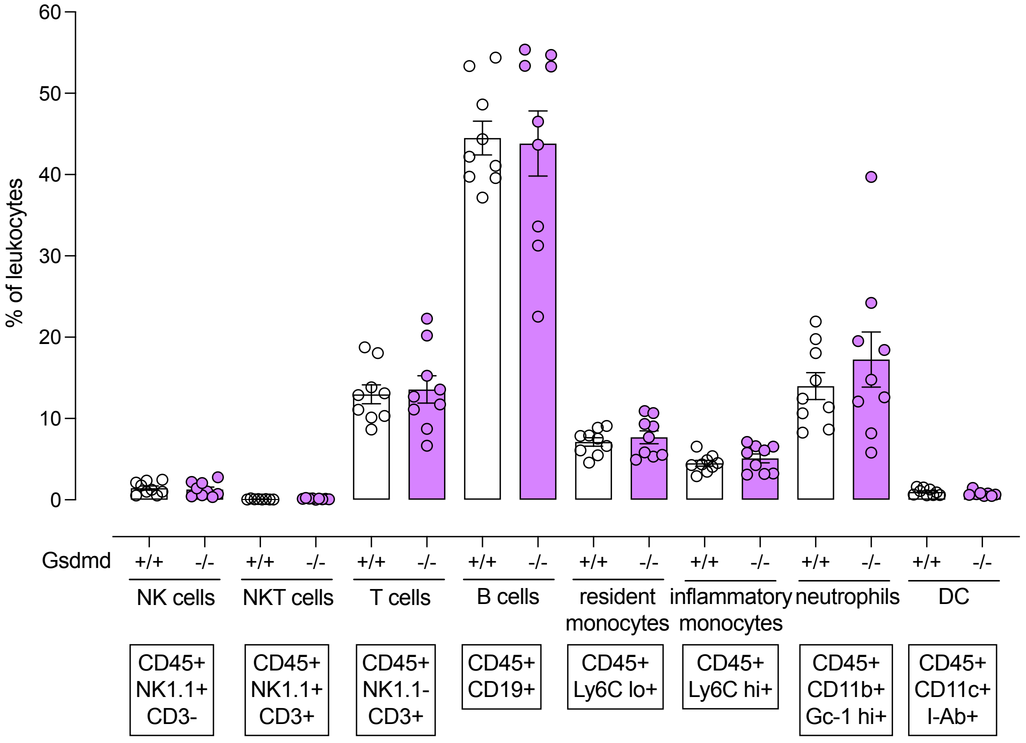

Analysis of blood leukocyte subsets. *ApoE$^{-/-}$ Gsdmd$^{-/-}$* and ApoE$^{-/-}$ Gsdmd$^{+/+}$ mice were fed a WD for 16 weeks. Blood samples were collected and leukocyte subsets were analyzed on a BD accuri C6 flow cytometer (n=9 mice per group).

Figure S3

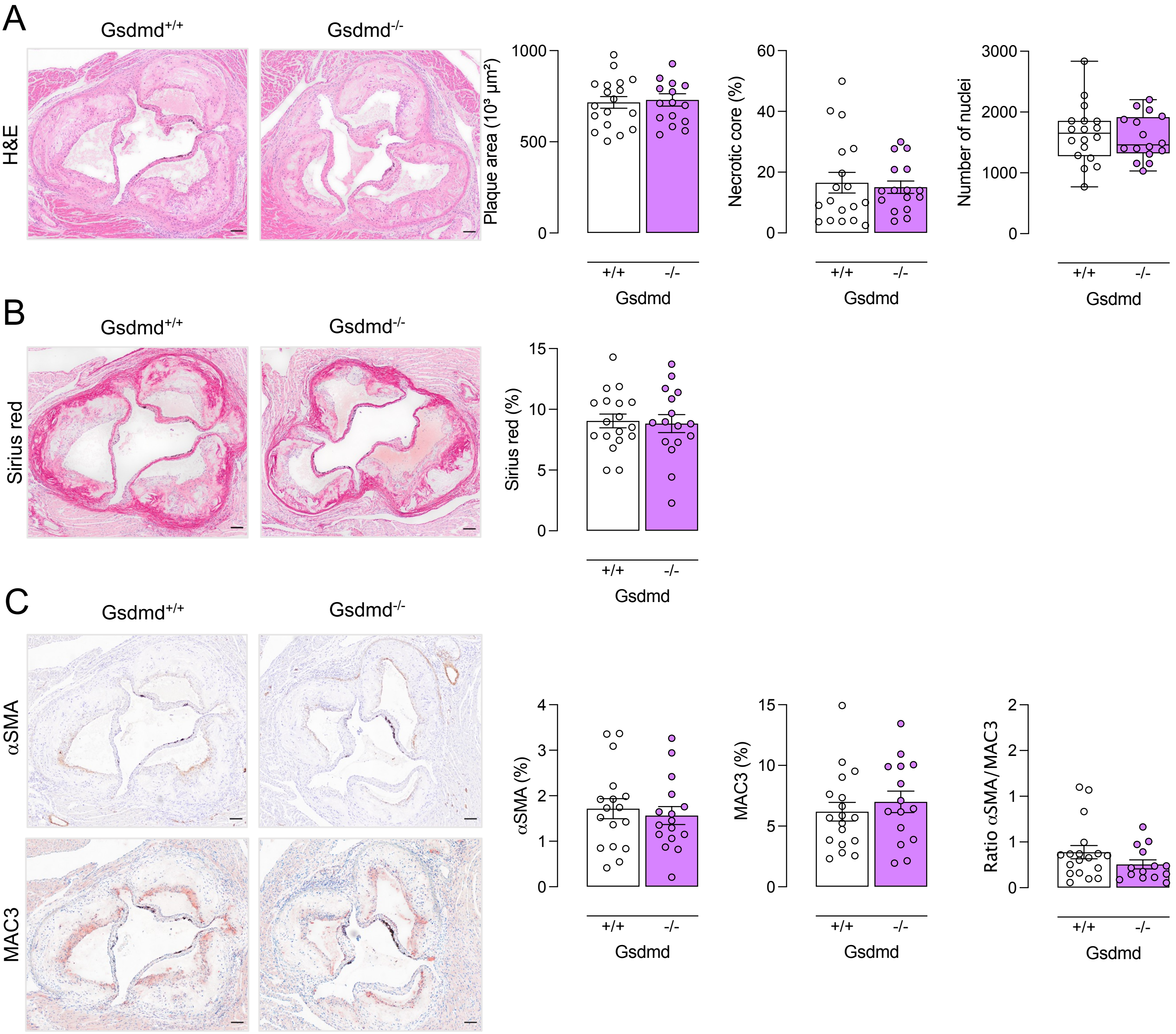

Plaque analysis in the aortic root from *ApoE$^{-/-}$ Gsdmd$^{-/-}$* and *ApoE$^{-/-}$ Gsdmd$^{+/+}$* mice. *ApoE$^{-/-}$ Gsdmd$^{-/-}$* and *ApoE$^{-/-}$ Gsdmd$^{+/+}$* mice were fed a WD for 16 weeks. Sections of the aortic root were stained with (A) hematoxylin/eosin to quantify plaque size, necrotic cores and cell infiltration, (B) Sirius red to measure total collagen content, (C) anti-MAC3 and anti-α-smooth muscle actin (αSMA) to determine macrophage and smooth muscle cell content, respectively, and to calculate the ratio of αSMA/MAC3 immunoreactivity. (independent samples t-test, boxplot: Mann-Whitney test, n=14-18 mice per group). Scale bar = 100 µm. Representative images are shown.
